# miRNA Regulation of T Cells in Islet Autoimmunity and Type 1 Diabetes

**DOI:** 10.1007/s11892-020-01325-9

**Published:** 2020-07-28

**Authors:** Martin G. Scherm, Carolin Daniel

**Affiliations:** 1 Institute of Diabetes Research, Group Immune Tolerance in Type 1 Diabetes, Helmholtz Diabetes Center at Helmholtz Zentrum München, Heidemannstrasse 1, 80939 Munich, Germany; 2 Deutsches Zentrum für Diabetesforschung (DZD), Ingolstaedter Landstrasse 1, 85764 Munich-, Neuherberg, Germany; 3 Division of Clinical Pharmacology, Department of Medicine IV, Ludwig-Maximilians-Universität München, 80337 Munich, Germany

**Keywords:** Immune regulation, Islet autoimmunity, Type 1 diabetes, Regulatory T cell, miRNA, Biomarker

## Abstract

**Purpose of Review:**

Regulatory T cells (Tregs) are critical contributors to immune homeostasis and their dysregulation can lead to the loss of immune tolerance and autoimmune diseases like type 1 diabetes (T1D). Recent studies have highlighted microRNAs (miRNAs) as important regulators of the immune system, by fine-tuning relevant genes in various immune cell types. In this review article, we discuss recent insights into miRNA regulation of immune tolerance and activation. Specifically, we discuss how the dysregulation of miRNAs in T cells contributes to their aberrant function and the onset of islet autoimmunity, as well as their potential as targets of novel intervention strategies to interfere with autoimmune activation.

**Recent Findings:**

Several studies have shown that the dysregulation of individual miRNAs in T cells can contribute to impaired immune tolerance, contributing to onset and progression of islet autoimmunity. Importantly, the targeting of these miRNAs, including miR-92a, miR-142-3p and miR-181a, resulted in relevant effects on downstream pathways, improved Treg function and reduced islet autoimmunity in murine models.

**Summary:**

miRNAs are critical regulators of immune homeostasis and the dysregulation of individual miRNAs in T cells contributes to aberrant T cell function and autoimmunity. The specific targeting of individual miRNAs could improve Treg homeostasis and therefore limit overshooting T cell activation and islet autoimmunity.

## Introduction

Type 1 diabetes (T1D) is an organ-specific autoimmune disease. Its high prevalence early in life and increasing incidence worldwide make it a considerable burden for the healthcare systems. The disease is characterized by the T cell-mediated destruction of the insulin producing beta cells in the pancreas, leading to impaired insulin secretion, hyperglycemia, and increased risk of both acute and chronic complications associated with significant morbidity and mortality [1]. FOXP3^+^ regulatory T cells (Tregs) are key players for the maintenance of immune homeostasis. Impaired Treg induction, stability and function are critically involved in the loss of immune tolerance and the activation of autoimmunity [2]. microRNAs (miRNAs) have been recently shown to contribute to immune function and homeostasis by controlling gene expression in various immune cell types, including Tregs. Targeting of miRNAs represents a promising approach to interfere with overshooting immune activation. In this review, we discuss the role of T cells for the onset and progression of islet autoimmunity and the complex interplay with miRNA regulatory networks. Furthermore, we address the therapeutic potential of miRNA targeting for future immunomodulatory strategies with the vision to interfere with autoimmunity.

## miRNA Basics and Biogenesis

Small non-coding RNAs (ncRNAs) have emerged as important contributors to the regulation of various biological processes including immune regulation, function and homeostasis. They are defined by their Argonaute (AGO) family protein-mediated mode of action and their length of 20–30 nucleotides. There are three distinct families of small ncRNAs with regulatory function: miRNA (microRNA), siRNA (small interfering RNA) and piRNA (PIWI-interacting RNA). The miRNA class is the most abundant in most tissues. miRNAs are ~22 nucleotides long, single-stranded and transcribed from the genome, alone or in polycistronic clusters. They control gene expression by complementary binding of their target mRNA, recruiting AGO family proteins and inducing mRNA cleavage, translational repression or mRNA deadenylation.

The recognition of the respective mRNA targets is facilitated by the miRNA seed sequence, a sequence which is specific for each miRNA, spanning from nucleotide position 2 to 7. While the seed sequence is crucial for target recognition and thus for the regulatory function of miRNAs, the binding of the mRNA target is additionally supported by nucleotides 8 and 13–16 of the miRNA [3]. There are groups of miRNAs, called miRNA clusters or families, which have highly similar seed sequences and substantially overlapping target genes [4]. Previous studies suggested that miRNA binding preferentially occurs in the 3′ untranslated region (UTR) [5], but more recent studies showed that miRNA binding sites are located at comparable levels in the 3’ UTR and the coding region of the mRNA targets, with only a slight preference for the 3’ UTR [6, 7•].

The biogenesis of miRNAs is a complex multi-stage process which takes place in the nucleus as well as the cytoplasm. It comprises the transcription of miRNA genes into primary miRNA (pri-miRNA) transcripts, processing into pre-miRNAs and subsequently into mature miRNAs. Pri-miRNAs are mainly transcribed by RNA polymerase II [8], and the primary transcript can contain a single miRNA or a cluster of multiple miRNAs [9]. The pri-miRNA is processed by Drosha [10] and DGCR8 [11, 12] and the resulting pre-miRNA by Dicer [13] to produce the mature miRNA duplex. One strand of the mature miRNA is loaded into AGO to form the miRNA-induced silencing complex (miRISC) and to guide the complex to its target mRNAs, while the other strand is discarded [14].

## Regulatory Role of miRNAs

The important role of miRNAs for various biological processes is illustrated by their complex regulatory networks. Each individual miRNA targets a multitude of genes and in addition most of these genes are targeted by more than one miRNA. Most human protein-coding genes have been shown to be miRNA targets with more than 60% containing multiple conserved miRNA binding sites and in addition even larger numbers of non-conserved sites [3–5]. So far, about 2600 mature human miRNA sequences are defined in the miRNA database MirBase [15]; the actual number of human miRNAs is thought to be much higher, as illustrated by the constant increase of newly defined miRNA sequences [16]. It has been shown that miRNAs harbor clear tissue-specific expression patterns, which are regulated at the transcription level and illustrate their important contribution to the regulation of tissue homeostasis and function [3, 17]. Given these complex regulatory networks and the broad role of miRNAs for the regulation of various biological processes, the dysregulation of miRNA expression can contribute to various diseases, including autoimmunity, cancer [18] and neurological diseases [19]. The first evidence for an important role of miRNAs in the regulation of the immune system dates back to 2004, when differential expression of several miRNAs was reported in specific immune cell subsets [20]. Since then, multiple studies clearly indicated the implication of miRNAs in various processes of immune regulation.

## Immune Regulation and T1D Autoimmunity

The clear discrimination between self and non-self is a critical requirement for the proper functioning of the immune system, which is detecting and eliminating harmful pathogens and distinguishing them from the organism’s own tissues. This delicate balance between immune activation and immune tolerance is a complex and tightly regulated process that requires precise control of lymphocyte development and function, with constant adjustments as required. The importance of proper immune regulation becomes evident when these control mechanisms are dysregulated, which leads to the development and activation of autoreactive lymphocytes and thereby is a critical contributor to autoimmunity. There are more than 80 diseases with an autoimmune etiology, which can be classified into systemic or organ-specific diseases, such as Multiple Sclerosis, Rheumatoid Arthritis and T1D, which is the most common autoimmune disease in young children [21]. The disease is characterized by impaired immune tolerance mechanisms, resulting in the immune-mediated destruction of pancreatic beta cells, diabetes symptoms and complications [22].

T1D progresses through distinct identifiable stages prior to the onset of the clinically overt disease [23, 24]. The appearance of autoantibodies against islet autoantigens defines the pre-symptomatic phase of T1D, which is therefore termed islet autoimmunity. During the last decades, several autoantigens have been identified, including insulin [25], glutamic acid decarboxylase (GAD) [26], insulinoma-antigen 2 (IA2) [27, 28] and zinc transporter 8 (ZNT8) [29]. Importantly, autoantibodies can be present in the blood many years before the onset of hyperglycemia [24, 30]. Despite ongoing research efforts and although recent studies significantly contributed to our understanding of T1D pathogenesis, the molecular mechanisms underlying the heterogeneity, the onset of islet autoimmunity and the progression to symptomatic diabetes remain poorly understood.

## Role of T Cells in T1D

The immune-mediated destruction of the insulin producing beta cells in the pancreatic islets of Langerhans is initiated by insulitis, a process of immune cell infiltration into the islets. The infiltrating immune cells consist of CD8^+^ T cells, CD4^+^ T cells, B cells and macrophages, with CD8^+^ T cells being the most abundant cell type [31]. Despite the high abundance of CD8^+^ T cells in the pancreatic infiltrates, various subsets of CD4^+^ T cells have been identified as important contributors to autoimmune diseases, including T1D, multiple sclerosis [32], rheumatoid arthritis [33] and Crohn’s disease [34]. In T1D, multiple studies have identified and further characterized infiltrating CD4^+^ T cells autoreactive against islet antigens. These identified subsets include Th1, Th2 [35], Th17 [36, 37], Th9, Th22 [38] and TFH cells [39, 40]. Although T cells clearly play a major role in the destruction of pancreatic beta cells, the underlying mechanisms promoting aberrant T cell activation in autoimmunity remain poorly understood.

Recent studies have focused on the contribution of specific T cell subsets to immune activation and autoimmunity and their findings point towards an important role of impaired function of Tregs [7•, 41–44]. Tregs are crucial contributors to the maintenance of immune homeostasis and their impaired induction, stability and function can critically contribute to the loss of immune tolerance and the activation of autoimmunity. They are characterized by the expression of CD4, CD25, and the transcription factor FOXP3, which is crucial for their development, maintenance and function [45, 46]. This is clearly illustrated by the deleterious consequences of mutation in the FOXP3 gene, resulting in fatal autoimmune phenotypes in both mice (scurfy mice) and humans (IPEX—immunodysregulation, polyendocrinopathy, enteropathy, X-linked syndrome) [47, 48]. In addition to their generation in the thymus, Tregs can be induced from naive CD4^+^ T cells in the periphery, most efficiently by the application of a strong-agonistic T cell receptor (TCR) ligand under subimmunogenic conditions [49–51].

## miRNAs as Biomarkers in T1D

Biomarkers provide an objectively quantifiable manner to characterize a disease. This enables both diagnosis and treatment as early and precisely as possible, and facilitates highly targeted and personalized interventions aimed at the maximum benefit for the patient. Consequently, the interest in biomarkers for studying diseases and developing novel treatment strategies continues to grow in many areas of clinical practice.

Levels of circulating miRNAs can be analyzed in small volumes of peripheral blood. Given this minimal invasiveness and the importance of miRNAs for immune homeostasis, several studies have investigated the impact of circulating miRNAs on disease development and their suitability to predict diseases progression [52–55]. miRNAs involved in the function of lymphocytes and beta cells were found to be differentially expressed in whole blood and serum obtained from newly diagnosed T1D patients compared to healthy controls. The abundance of miR-21a and miR-93, which are involved in NF-κB signaling and negatively regulate apoptotic and inflammatory genes, was significantly reduced in peripheral blood mononuclear cells (PBMCs) of T1D patients [53]. In contrast, residual beta cell function and glycemic control in individuals with recent onset of T1D were associated with high levels of miR-25 [52]. miR-146a was significantly downregulated in PBMCs of patients with newly diagnosed T1D and the decreased miRNA expression was associated with high GAD autoantibody titers in the serum [56], while an upregulation of miR-326 correlated with the presence of autoantibodies against GAD and IA2 [54]. A meta-analysis of studies investigating circulating miRNA profiles in T1D patients revealed eleven differentially expressed miRNAs: miR-21-5p, miR-24-3p, miR-100-5p, miR-146a-5p, miR-148a-3p, miR-150-5p, miR-181a-5p, miR-210-5p, miR-342-3p, miR-375 and miR-1275. These miRNAs are involved in immune regulation, cell proliferation and insulin processing, making them potential biomarkers for T1D [57].

Despite the importance of the pre-symptomatic phase of T1D only limited studies have investigated miRNA levels in individuals with islet autoimmunity before the onset of clinical T1D. One of these rare studies found similar miRNA levels in the serum of high-risk individuals positive for multiple islet autoantibodies and healthy controls and no specific miRNA signature could be identified in the high-risk group. Interestingly, several miRNAs were associated with glucose homeostasis and autoantibody titers and these miRNAs correlated with glycemic status and ongoing islet autoimmunity in high-risk individuals [58]. Another study investigated miRNAs in the serum of 150 autoantibody-positive and 150 autoantibody-negative family-matched siblings and found several miRNAs in the serum reflecting islet autoimmunity and progression to T1D with miR-21-3p, miR-29a-3p and miR-424-5p showing the strongest correlation [55]. Despite these important insights, the full potential of circulating miRNAs for the assessment of T1D pathogenesis and progression is still limited due to: (1) In organ-specific diseases like T1D, profiles from whole blood or serum might not reflect the relevant changes in the affected organ or cell type. (2) Relevant changes in miRNA profiles might result from changes in the miRNA profile or the abundance of specific immune cell populations, rather than global changes in the miRNA expression. (3) Changes in miRNA abundance might result from the loss of glucose homeostasis and therefore not be related to disease pathogenesis. To overcome these limitations and to increase the explanatory power of circulating miRNAs identified in the abovementioned studies, the analysis of specific miRNAs in cell types which are directly involved in the activation and progression of autoimmunity gets into the focus.

## miRNA Regulation of Regulatory T Cells

In line with their regulatory role in various biological settings, including the immune system, miRNAs have also been highlighted as direct modulators of Tregs. The Treg-specific interference with the miRNA processing machinery in mice had no effect on thymic development of Tregs, but it resulted in reduced suppressive function of Tregs and deleterious systemic autoimmunity [59••, 60••, 61, 62]. Based on these findings, multiple studies investigated the underlying mechanisms of these defects and specifically the contribution of individual miRNAs. These studies revealed miRNA regulation of thymic Treg development, Treg induction, FOXP3 expression, Treg stability and suppressive function (Table 1).

**Table 1:** miRNAs involved in Treg induction, maintenance and function

miRNA	miRNA regulation	miRNA target	effect on Tregs	organism	target validation		miRNA effect validation		reference
					indirect	direct	in vitro	in vivo	
let7i	up	IGF1R	decreased Treg induction	human	X		X		[63]
miR10a	down	IFNγ	increased Foxp3 abundance	mouse		X	X	X	[64]
miR15a/16	up	Foxp3	decreased Foxp3 abundance	human		X	X	–	[65]
miR15b/16	up	Rictor, mTor	increased Treg induction	mouse	X		X	X	[66]
miR17	up	IKZF4	decreased Treg frequencies	mouse	X		X	X	[67]
miR19b	up	PTEN	decreased Treg frequencies	mouse	X		X	X	[67]
miR21	up	unknown	increased Foxp3 abundance	human	–	–	X	–	[68]
miR21	down	STAT3	decreased Treg frequencies	human	X		–	–	[69]
miR24	down	Foxp3	increased Foxp3 abundance	human		X	–	–	[70]
miR25	up	TGFB signaling	decreased suppression	human		X*	–	–	[71]
miR31	down	Foxp3	increased Foxp3 abundance	human		X	X	–	[68]
miR92a	up	KLF2 (…)	decreased Treg induction	human/mouse		X	X	X	[42]
miR95	up	unknown	increased Foxp3 abundance	human	–	–	–	–	[70]
miR99a	up	mTor	increased Treg induction	mouse		X	X	X	[72]
miR100	up	SMAD2/3	decreased Treg induction	human	X		X		[73]
miR125a-5p	down	CXCL13	decreased Treg frequencies	human		X*	–	–	[74]
miR126	down	p85B	decreased Treg induction	human/mouse	X		X	X	[75]
miR142-3p	up	Tet2	decreased Treg induction and stability	human/mouse		X	X	X	[7]
miR146a	down	STAT1	decreased suppression	human		X*	–	–	[76]
miR146a	down	STAT1	decreased suppression	mouse		X	X	X	[77]
miR146b	up	TRAF6	decreased suppression	human		X	X	X	[78]
miR150	up	mTor	increased Treg induction	mouse		X	X	X	[72]
miR181a	up	PI3K signaling	decreased Treg induction	human/mouse		X	X	X	[44]
miR182	up	Foxo1	decreased Treg frequencies	mouse	X		X	X	[79]
miR200a	up	unknown	decreased Treg frequencies	human	–	–	–	–	[80]
miR210	down	Foxp3	increased Foxp3 abundance	human		X	–	–	[70]
miR210	up	Foxp3	decreased Treg frequencies	human		X	X	–	[81]
miR214	up	PTEN	increased Treg frequencies	mouse	X		X	X	[82]
miR326	up	Ets-1	decreased Treg frequencies	human	X		–	–	[83]
miR663	up	TGFB1	decreased Treg frequencies	human/mouse		X	X	X	[84]

*shown previously

The expression of miR-10a is restricted to Tregs following the exposure to retinoic acid and the abundance of this miRNA correlates with a low susceptibility to autoimmune diseases in mice [85, 86]. miR-10a stabilizes the Treg-specific gene signature by targeting several effector T cell genes including *Bcl6* and *Ncor2*. However, the functional targets of miR-10a may be targeted by other miRNAs as well, because miR-10a deficiency alone does not result in impaired Treg function or autoimmunity. The analysis of miRNA expression in Tregs revealed several differentially expressed miRNAs with both up- and downregulation compared to CD4^+^CD25^−^ T cells. Interestingly, miR-31 which was downregulated in Tregs, directly targets the 3’UTR of *Foxp3* mRNA [68]. This predicted binding was experimentally confirmed using lentiviral transduction of T cells, which resulted in reduced FOXP3 levels. In addition, miR-21, which was highly expressed in Tregs, is a positive, though indirect, regulator of FOXP3 expression. miR-155 is highly abundant in Tregs and directly regulated by FOXP3. In mouse models miR-155 deficiency resulted in impaired Treg development and homeostasis and consequently reduced levels of Tregs in the thymus and the spleen [87, 88]. In miR-155-deficient Tregs the expression of FOXP3 is reduced and instable while in vitro Treg induction is unaffected. miR-155 targets suppressor of cytokine signaling 1 (SOCS1), a negative regulator of STAT5 signaling which determines the responsiveness to IL-2, a critical regulator of Treg homeostasis [89]. However, miR-155-deficient Tregs can prevent autoimmune diseases in mice, indicating that miR-155 is crucial for FOXP3 expression and Treg stability but does not directly affect their suppressive function [87].

The efficient in vitro Treg induction is also subject to regulation by miRNAs. In vitro Treg induction experiments using both Dicer and Drosha deficient naive CD4^+^ T cells resulted in a significantly reduced expression of FOXP3 in induced Tregs compared to wild type naive CD4^+^ T cells [59••, 60••]. Interestingly, miRNAs with both positive and negative regulatory effects on in vitro Treg induction have been identified in a miRNA screen [90] and several miRNAs form networks to cooperatively regulate Treg induction. For example, miR-150 induced reduction of mTOR occurs only in presence of miR-99a and a similar cooperation has been shown for miR-15a-16 and 15b-16. Another miRNA targeting the PI3K/Akt/mTOR pathway is miR-126. It targets p85β, which is a regulatory subunit of PI3K, reducing PI3K/Akt/mTOR pathway activity and favoring Treg induction. By contrast, miR-126 inhibition increases the activity of the PI3K/Akt/mTOR pathway, inhibiting FOXP3 expression and impairing Treg induction [75]. miR-155 also contributes to proper Treg induction in vitro by targeting *Socs1* which is in line with its role for thymic Treg generation [91]. As mentioned above, the miRNA screen also revealed miRNAs with a negative effect on Treg induction in vitro [90]. Two members of the miR-17 ~ 92 cluster, miR-17 and miR-19, negatively regulate Treg induction while they have no impact on thymic Treg development [92]. miR-17 has been shown to directly target the cAMP-responsive element binding protein 1 (*Creb1*) and the TGFβ-receptor II, both involved in proper Treg induction. The TGFβ signaling pathway is also a target of the miR-23-miR-27-miR-24 cluster, and consequently a high abundance of this cluster impairs Treg generation [93].

## Mechanistic Dissection of miRNAs in T Cells during Autoimmunity

As illustrated above, several recent studies illustrated the importance of miRNA mediated regulation for immune homeostasis by controlling the accurate function of various immune cell subsets, including Tregs. While this makes them of considerable importance for the field, the underlying, relevant pathways ultimately triggering insufficient immune tolerance and the onset of T1D autoimmunity, remain insufficiently understood. Given the importance of proper Treg function, induction and stability, three recent studies focused on the direct interplay between the dysregulated expression of individual miRNAs in T cells during the onset of autoimmunity and impaired Treg induction from naive CD4^+^ T cells.

## miR-142-3p

**Fig. 1:**
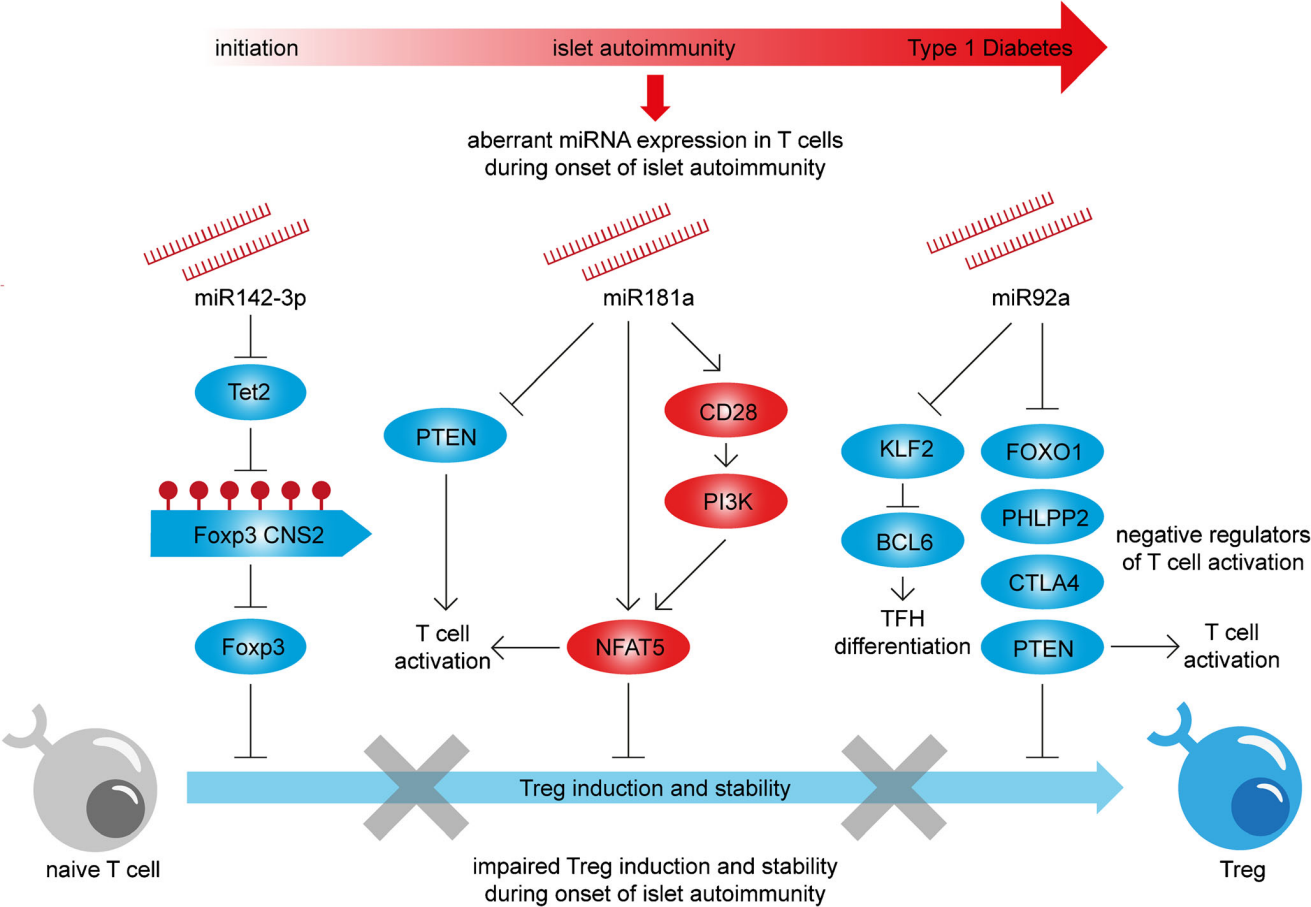
Role of T cell specific miRNAs in autoimmune activation. During the onset of islet autoimmunity high levels of miR-142-3p, miR-181a and miR-92a and their downstream pathways contribute to enhanced T cell activation and impairments in Treg induction from naive CD4^+^ T cells

The first study showed that during islet autoimmunity increased miR-142-3p expression leads to impaired epigenetic remodeling and consequently interferes with the efficient induction of Tregs and Treg stability (Fig. 1). Specifically, the upregulation of miR-142-3p during the onset of islet autoimmunity resulted in decreased expression of its direct target, the methylcytosine dioxygenase Tet2, which is a mediator of DNA demethylation. By catalyzing DNA demethylation of regulatory regions and consequently altering the accessibility of the DNA to transcription factors, Tet enzymes are involved in the regulation of various cellular processes. They’re involved in the differentiation of CD4^+^ T cells in humans and mice, as well as Treg homeostasis and function by active demethylation of the *FOXP3* conserved noncoding sequence 2 (CNS2), which ensures long-term stability of FOXP3 expression in Tregs [94–97]. The miR-142-3p mediated reduced abundance of the epigenetic modifier Tet2 was directly linked to impaired DNA demethylation at the *FOXP3* CNS2 locus during islet autoimmunity and led to reduced frequencies of FOXP3^+^ Tregs in the pancreas of mice with ongoing islet autoimmunity. The inhibition of miR-142-3p restored Tet2 levels, improved Treg induction and stability in vitro and reduce islet autoimmunity in mouse models of islet autoimmunity in vivo. As a next step, the relevance of these findings for established human T1D and their translatability was shown in humanized mouse models. Furthermore, and in line with the complex regulatory networks targeted by individual miRNAs, the study revealed multiple miR-142-3p targets with important roles in Treg development and function. Specifically, *Stat5*, *Smad3* and Tgfb receptors (T*gfbr*1, T*gfbr*2 and T*gfbr*3) were identified as potential targets. Stat5 is a downstream target of IL-2 involved in Treg development [98]; TGFβ exhibits potent immunoregulatory properties, including the expression of FOXP3 in Tregs via phosphorylation of Smad proteins [99]. Both signaling pathways add to the identified mechanism of Tet2-mediated DNA demethylation to ensure FOXP3 expression and maintenance and consequently proper Treg function and stability. These findings suggest that miR-142-3p targets a regulatory network of genes involved in the regulation of Treg function and stability; such a broad impact helps explain how the targeting of a single miRNA has such a considerable effect on Treg homeostasis in a highly complex setting like autoimmunity. In addition, Tet2 catalyzes the demethylation of regulatory regions of various genes. This study showed the importance of Tet2-mediated demethylation of the *FOXP3* CNS2 for Treg function and stability in the context of autoimmunity. However, additional regulatory regions might be controlled by Tet2 and contribute to the regulation of FOXP3. These results offer a new mechanistic model where during islet autoimmunity miRNA142-3p/Tet2-mediated impairments in Treg induction and stability contribute to the activation and progression of islet autoimmunity. Moreover, specific targeting of miR-142-3p or Tet2 might contribute to the development of novel intervention strategies, aiming at improved Treg induction and stability to interfere with the onset of islet autoimmunity.

## miR-181a

In a second study, excessive T cell activation was linked to differential miRNA expression during the onset of islet autoimmunity (Fig. 1). The excessive T cell activation was found in children with recent onset of islet autoimmunity and resulted in enhanced frequencies and proliferation of FOXP3^int^CD4^+^ T cells, which interferes with efficient Treg induction [43, 44]. Specifically, this increased T cell activation could be directly linked to higher levels of miR-181a, which reportedly alters signaling thresholds in T cells by modulating the sensitivity to antigenic stimulation [100]. The phosphatase PTEN, which is a negative regulator of T cell activation, was identified as a direct target of miR-181a and PTEN inhibition resulted in increased PI3K signaling and activation of NFAT5. NFAT5 plays an important role for T cell activation and its abundance was increased as a result of high miR-181a levels. Furthermore, elevated miR-181a levels increased the expression of the costimulatory molecule CD28 which is also involved in the activation of the PI3K pathway [101] and consequently T cell activation. In line with these findings, the inhibition of miR-181a improved Treg induction from naive CD4^+^ T cells, while a miR-181a mimic resembled the impaired Treg induction observed during recent onset of islet autoimmunity. These findings were confirmed in mouse models, including those of T1D [44•]. Specifically, high levels of miR-181a and NFAT5, decreased abundance PTEN and reduced Treg induction capacity from naive CD4^+^ T cells were found in NOD mice with islet autoimmunity. The in vivo inhibition of miR-181a in those mice reduced islet autoimmunity, decreased CD28 and NFAT5 expression and enhanced PTEN levels. Treg induction was also improved by pharmacological inhibition of NFAT5, and in *Nfat5* deficient mice where increased levels of PTEN were also observed.

## miR-92a

The third study provided evidence that high levels of miR-92a during the onset of islet autoimmunity mediate the induction of T follicular helper (TFH) cell precursors as well as impaired Treg induction from naive CD4^+^ T cells, two mechanisms that are likely involved in the onset and progression of islet autoimmunity [42•] (Fig. 1). TFH cells, which are a subset of CD4^+^ T cells, provide help to B cells to produce high-affinity antibodies, making them crucial contributors to humoral immunity [102–104]. This important role for antibody production suggests TFH precursors as an important effector T cell subset in autoimmunity. Both insulin-specific and polyclonal TFH precursors were increased in children with recent onset of islet autoimmunity [42•]. Analysis of differential miRNA expression in these children identified miR-92a as upregulated and there was a positive correlation between miR-92a expression and the frequencies of TFH precursor cells in the blood. In experimental in vitro TFH induction assays, the inhibition of miR-92a reduced TFH induction, while a miR-92a mimic had the opposite effect [42•]. This was in line with the identification of several negative regulators of T cell activation including *Ctla4*, *Foxo1* and *Pten* as targets of miR-92a. In addition, *Klf2* was identified as a novel target of miR-92a and the miR-92a mediated downregulation of *Klf2* was essential for the induction of TFH precursor cells. The decreased levels of PTEN resulted in the activation of the PI3K pathway which limits efficient Treg induction. Consequently, in vitro Treg induction was impaired in the presence of a miR-92a mimic, and high miR-92a levels in children with recent onset of islet autoimmunity were accompanied by reduced frequencies of insulin-specific Tregs. Conversely, PI3K signaling promoted TFH cell induction, since the effect of a miR-92a mimic is reduced in presence of a PI3K inhibitor and increased when PTEN signaling is inhibited.

In addition to the studies described above, miR-92a and miR-181a are also involved in the pathogenesis of other autoimmune conditions, including lymphoproliferation, Th17-mediated inflammation and autoimmune neuroinflammation [67, 105, 106].

## Targeting of T Cell miRNAs and Clinical Relevance

The studies described above illustrate mechanisms by which increased miRNA expression directly contributes to autoimmune activation and progression. These findings, as well as the general importance of miRNAs for the precise regulation of the immune system, suggest that targeting miRNAs could represent an innovative intervention strategy to improve Treg induction and stability to interfere with islet autoimmunity. The relatively simple and well-known structure of miRNAs makes them attractive drug targets. This enables the efficient targeting of miRNAs using highly specific inhibitors, while mimics can be used to increase their activity, facilitating the fine-tuning of the regulation of their target genes. The complex regulatory networks of miRNAs, with each miRNA targeting a multitude of genes, hinder the direct and precise control of individual genes. However, these complex regulatory networks provide a potential explanation of the considerable effect of individual miRNAs in various settings, including their involvement in disease pathogenesis.

In autoimmunity and T1D miRNA-targeting agents were successfully used in vitro, in mouse models of autoimmune disease, and in humanized mice, resulting in upregulation of the respective miRNA targets, improvements in Treg function and reduced islet autoimmunity [7•, 42, 44]. Furthermore, several clinical trials are currently investigating miRNA-based therapeutics for the treatment of hepatitis C virus infection, Alport syndrome, fibrosis, lymphoma, leukemia, inflammatory bowel disease, liver cancer and wound healing (summarized in: [107, 108]). To ensure both efficacy and safety of miRNA-targeting drugs, these approaches require the selective and targeted delivery of miRNA inhibitors to the desired cell population, which remains challenging [109]. This is particularly important for the efficient treatment of organ-specific autoimmune diseases such as T1D but also for other disease settings which require a precise and local intervention in affected cell types. Several recent approaches resulted in considerable advances regarding the uptake of miRNA targeting agents by immune cells, as well as their targeted delivery. The use of oligonucleotide encapsulation techniques and nanoparticles led to improved uptake by immune cells and combinations of antibodies and nanoparticles are an important step towards the precise targeting of T cells or even T cell subsets [110, 111]. However, the design and validation of future treatment approaches will require the development of additional targeted strategies for T cell-specific targeting of miRNAs as well as optimized humanized mouse models to mimic the mechanisms underlying human autoimmune diseases in a preclinical model.

## Conclusion

In order to better understand the molecular mechanisms promoting the onset of autoimmunity and the progression to T1D, the contribution of individual T cell subsets and how these subsets are regulated are of great importance. miRNAs are emerging as critical contributors to immune function and homeostasis, and they fine-tune the expression of relevant genes in various immune cell types, including Tregs. Therefore, recent studies focused on the contribution of individual miRNAs to aberrant T cell function in the setting of autoimmunity and described mechanisms by which miRNA levels impact autoimmune activation and progression. These insights suggest that the targeted and specific modulation of miRNA levels has the potential to reestablish Treg function and stability and in turn interfere with autoimmunity in future intervention strategies.
